# Dynamics of Defense Responses and Cell Fate Change during Arabidopsis-*Pseudomonas syringae* Interactions

**DOI:** 10.1371/journal.pone.0083219

**Published:** 2013-12-11

**Authors:** Safae Hamdoun, Zhe Liu, Manroop Gill, Nan Yao, Hua Lu

**Affiliations:** 1 Department of Biological Sciences, University of Maryland Baltimore County, Baltimore, Maryland, United States of America; 2 State Key Laboratory of Biocontrol, Guangdong Key Laboratory of Plant Resources, School of Life Sciences, Sun Yat-sen University, Guangzhou, P.R. China; Virginia Tech, United States of America

## Abstract

Plant-pathogen interactions involve sophisticated action and counteraction strategies from both parties. Plants can recognize pathogen derived molecules, such as conserved pathogen associated molecular patterns (PAMPs) and effector proteins, and subsequently activate PAMP-triggered immunity (PTI) and effector-triggered immunity (ETI), respectively. However, pathogens can evade such recognitions and suppress host immunity with effectors, causing effector-triggered susceptibility (ETS). The differences among PTI, ETS, and ETI have not been completely understood. Toward a better understanding of PTI, ETS, and ETI, we systematically examined various defense-related phenotypes of Arabidopsis infected with different *Pseudomonas syringae* pv. *maculicola* ES4326 strains, using the virulence strain DG3 to induce ETS, the avirulence strain DG34 that expresses avrRpm1 (recognized by the resistance protein RPM1) to induce ETI, and HrcC^-^ that lacks the type three secretion system to activate PTI. We found that plants infected with different strains displayed dynamic differences in the accumulation of the defense signaling molecule salicylic acid, expression of the defense marker gene *PR1*, cell death formation, and accumulation/localization of the reactive oxygen species, H_2_O_2_. The differences between PTI, ETS, and ETI are dependent on the doses of the strains used. These data support the quantitative nature of PTI, ETS, and ETI and they also reveal qualitative differences between PTI, ETS, and ETI. Interestingly, we observed the induction of large cells in the infected leaves, most obviously with HrcC^-^ at later infection stages. The enlarged cells have increased DNA content, suggesting a possible activation of endoreplication. Consistent with strong induction of abnormal cell growth by HrcC^-^, we found that the PTI elicitor flg22 also activates abnormal cell growth, depending on a functional flg22-receptor FLS2. Thus, our study has revealed a comprehensive picture of dynamic changes of defense phenotypes and cell fate determination during Arabidopsis-*P. syringae* interactions, contributing to a better understanding of plant defense mechanisms.

## Introduction

Plants have evolved sophisticated defense systems to recognize pathogens and subsequently restrict their invasion. Pathogen-associated molecular patterns (PAMPs) are conserved molecules or structures that are present in a group of similar microbes. Plants use cell surface receptors called pattern recognition receptors (PRRs) to recognize PAMPs as non-self and subsequently activate PAMP-triggered immunity (PTI), a basal defense to prevent further pathogen colonization in plants [1–3]. The best-studied PRR in Arabidopsis is FLAGELLIN SENSING 2 (FLS2) that directly binds bacterial flagellin and activates defense signaling involving MAPK cascade [4,5]. Successful pathogens can suppress PTI with effector proteins, which in bacterial pathogens are secreted via the type three secretion system (TTSS) to the host cells [6]. Such defense suppression leads to effector-triggered susceptibility (ETS) in the host. However, when a pathogen effector is recognized by a cognate host resistance (R) protein, much stronger defense, termed effector-trigged immunity (ETI) or R-gene mediated defense, is activated. ETI can lead to systemic acquired resistance, a form of enhanced disease resistance against a broad-spectrum of pathogens with long-lasting effects at the whole plant level [7,8]. 

During different layers of defense responses, host plants often undergo global transcriptional reprogramming [9–13]. A careful microarray analysis with RNA isolated from Arabidopsis infected with different *Pseudomonas syringae* strains to induce PTI, ETS, or ETI has revealed that there are quantitative and kinetic differences in gene expression during PTI, ETI, and ETS [10]. Besides transcriptional reprogramming, PTI, ETS, and ETI also involve the induction of various signaling molecules and the activation of programmed cell death. For instance, salicylic acid (SA) is the small phenolic compound critical for defense signaling and SA accumulation is induced significantly upon pathogen infection. Reducing SA levels, using mutants impaired in SA biosynthesis, such as the *SA induction-deficient 2*/*enhanced disease* susceptibility *16* (*sid2/eds16*) mutants [14], and/or blocking SA signaling, such as the *nonexpressor of* pr *genes 1-1* (*npr1-1*) mutant [15–17], compromise plant disease resistance. Exogenous applications of SA agonists, such as benzo (1–3) thiadiazol-7-carothioic acid (BTH), confer enhanced disease resistance in plants [18–20]. Oxidative bursts are also induced during PTI, ETS, and ETI, leading to production of reactive oxygen species (ROS). ROS can signal defense responses, cause cross-linking to strengthen cell wall, and at high concentrations directly kill pathogens as well as host cells [21,22]. Host cell death is commonly induced during an infection process. The hypersensitive response, a typical ETI during which host R proteins recognize cognate pathogen effectors, is characterized by massive cell death in the local infected region to quickly deprive pathogens of water and nutrients and thereby to kill the pathogens. 

Although many prior studies have tested accumulation of SA and ROS and the induction of cell death as part of defense phenotype assays of pathogen-challenged plants, how these signaling molecules and cell death formation change at different time points during PTI, ETS, and ETI, has not been compared under the same experimental condition. Similar to global gene expression profiling [10], a detailed analysis of the behavior of these defense signaling molecules and cell death formation in plants upon pathogen attack should contribute to a better understanding of PTI, ETS, and ETI, thereby host defense mechanisms. 

In this report, we carefully examined several defense related phenotypes in a time course during PTI, ETS, and ETI, using Arabidopsis-*P. syringae* as a model system. We found that there are dynamic differences between PTI, ETS, and ETI in SA accumulation, expression of the defense marker gene *PR1*, and cell death formation. Such differences are dependent on the doses of the strains used. In addition, our data provide precise temporal and spatial information on H_2_O_2_ during PTI, ETS, and ETI. Together these data support that the differences between PTI, ETS, and ETI are both quantitative and qualitative. Interestingly, we observed abnormal growths in the leaves at late infection stages, most obviously during PTI. The abnormal growths contain enlarged cells that have increased nuclear DNA content, suggesting a possible activation of endoreplication in host cells by *P. syringae* infection. Such hypertrophy of host cells induced by pathogen infection has been reported in several other plant pathosystems [23–27] but has never been shown during Arabidopsis-*P. syringae* interactions. Thus, our study has demonstrated a comprehensive picture of dynamic changes of defense phenotypes and cell fate determination during Arabidopsis-*P. syringae* interactions, contributing to a better understanding of plant defense mechanisms. 

## Materials and Methods

### Plant materials

All Arabidopsis plants used on this paper were in Columbia-0 (Col-0) background and were grown in growth chambers with a 12hr light/12hr dark cycle, light intensity at 200µmol m^-2^ s^-1^, 60% humidity, and 22 °C. The mutants *fls2-1*, *sid2-1*, and *npr1-1* were previously described [28]. 

### *Pseudomonas syringae* infection

*Pseudomonas syringae* pv. *maculicola* ES4326 strains DG3 (DG3), DG34 (expressing the avirulence effector *avrRpm1*), and HrcC^-^ (a TTSS deficient mutant that cannot deliver effectors) were described previously [29–31]. Bacterial culture and preparation were conducted as described [32]. The fourth to sixth leaves of 30-day-old plants were infiltrated with *P. syringae* strains at the indicated concentrations, using a 1 mL needleless syringe, and were collected at the appropriate times for further analyses. 

### RNA analysis

Leaves of 30-day-old plants infected with *P. syringae* were harvested for RNA extraction followed by northern blotting as described [33]. Radioactive probes were made by PCR, using an antisense primer specific for a gene fragment in the presence of [^32^P] dCTP. Primers for *PR1* were described previously [34]. 

### SA measurement

Free and total SA (glucosylated SA) were extracted from leaves of 30-day-old plants infected with *P. syringae* and quantified with an HPLC instrument as previously described [28,33].

### Cell death staining

Infected leaves were stained with trypan blue for visualization of cell death, according to Ng et al [33]. Photographs of the stained leaves were taken with a CCD camera (Cool Snap HQ^2^ , Photometrics, USA) connected to a dissecting microscope (Leica M205 FA, Leica Microsystems, Germany). At least four leaves from four plants of each treatment were stained and examined for cell death.

### Analysis of leaf morphology with light microscopy

The fourth to sixth leaves of 30-day-old plants were infiltrated with *P. syringae* strains at the indicated concentrations, using 10 mM MgSO_4_-infiltrated leaves as a control. For flg22 (GenScript USA Inc.) or BTH (a kind gift from Robert Dietrich (Syngenta)), leaves were infiltrated with flg22 (1 μM or 10 μM) or sprayed with BTH (10 μM or 300 μM), using water-treated leaves as a control. For quantification of abnormal growths, at least 25 leaves from 12 plants were used for each BTH treatment or bacterial infection and 18 leaves from 7 plants were used for each flg22 treatment. To examine leaf cross sections, infiltrated leaves were cut into 2x4 mm sections, using at least six sections from six plants in each treatment. The sections were fixed in a solution containing 1% OsO_4_ and embedded in LR White resin, according to manufacturer's instructions (Electron Microscopy Sciences, PA). One-micron sections were cut with an ultra-microtome (Reichert-Jung Ultracut E, Austria), stained with 1% toluidine blue O as described [35,36], examined and photographed using a CCD camera connected to a Leica dissecting microscope. 

### Nuclear DNA quantitation by DAPI staining

Leaves infected with *P. syringae* strains, or treated with flg22, BTH, or mock solutions were cut into 3x6 mm sections, using at least six sections from six plants for each treatment. The sections were fixed in a solution containing 75% ethanol and 25% acetic acid and embedded in paraplast (McCormic Scientific, IL). Fifteen-micron sections were cut and stained with 4’, 6-diamidino-2-phenylindole (DAPI) (Fluoromount-G^TM^, Cat. No. 0100-20, SouthernBiotech, AL). Images of nuclei were captured with a constant exposure time, using a DS cooled camera head (DS-Fi1c, Nikon, Japan) attached to a compound microscope (Nikon Eclipse E200, Nikon, Japan). The relative fluorescence unit (RFU) of a nucleus was quantified by subtracting the background fluorescence from the fluorescence of the nucleus, using ImageJ (Version 1.45s). As a reference, the average RFU of guard cells was set as 2C as previously described [24,36,37]. The relative nuclear DNA content of non-guard cells was calculated as following: RFU of a non-guard cell/RFU of guard cells x 2C. At least 60 nuclei were used for each data point. 

### H_2_O_2_ localization by cerium staining

To detect the precise localization of H_2_O_2_, we used cerium staining as described previously [38,39]. Briefly, the fourth to sixth leaves of 30-day-old plants were infiltrated with *P. syringae* strains at OD_600_ 0.01, using 10 mM MgSO_4_ treatment as a control. Infected leaves from six plants were cut into 1 x 2 mm pieces and incubated with freshly prepared 5 mM CeCl_3_ in 50 mM 3-(N-morpholino) propanesulfonic acid (MOPS) at pH 7.2 for 1 h. A duplicate set of samples was incubated with the solution without CeCl_3_ as controls. The samples were then prefixed with 2.5% (v/v) glutaraldehyde and 2% (w/v) paraformaldehyde in 0.1 M cacodylate buffer (pH 7.2) followed by a post-fixation with 1% (v/v) osmium tetroxide for 1 h. The fixed samples were dehydrated in serial concentrations of ethanol and embedded in Epon 812 resin (Electron Microscopy Sciences). Ultra-thin sections (90 nm) were cut and subsequently collected on copper grids (200 mesh). At least six sections from each sample were observed and photographed with a transmission electron microscope (JEOL JEM-1400) at an accelerating voltage of 120 kV.

### Statistical analysis

Statistical analyses were performed with one-way ANOVA Fisher’s protected least significant difference (PLSD) tests (Statview 5.0.1). P values less than 0.05 were considered to be significantly different among samples. Data were presented as means ± standard deviation. All experiments were repeated at least two times with similar results.

## Results

### Quantitative and kinetic differences in SA accumulation and PR1 expression during PTI, ETS, and ETI

To systematically examine the differences in defense phenotypes among PTI, ETS, and ETI, we infected Arabidopsis plants with *P. syringae* pv. *maculicola* ES4326 strains. Strain DG3 is virulent and can induce ETS while strain DG34 expresses the avirulent effector avrRpm1 recognized by the R protein RPM1 and can induce ETI [30,40]. Strain HrcC^-^ is TTSS-defective and does not deliver effectors into host cells [31,41]. HrcC^-^ has been used as a natural pathogen to activate PTI [42–44]. We used two concentrations for each strain to challenge wild type Col-0, OD_600_ 0.01 (10^7^ colony forming unit (CFU)/ml) and 0.001 (10^6^ CFU/ml) for DG3 and DG34 while OD_600_ 0.1 (10^8^ CFU/ml) and 0.01 for HrcC^-^. We did not use HrcC^-^ at OD_600_ 0.001 because this concentration did not induce detectable defense phenotypes in our preliminary study. The infected leaves were harvested in a 48-hour time course for SA quantification and expression analysis of the defense marker gene *PR1* [30]. Since both free and total SA levels are relevant for defense [33], we measured both SA levels with these samples.

**Figure 1:**
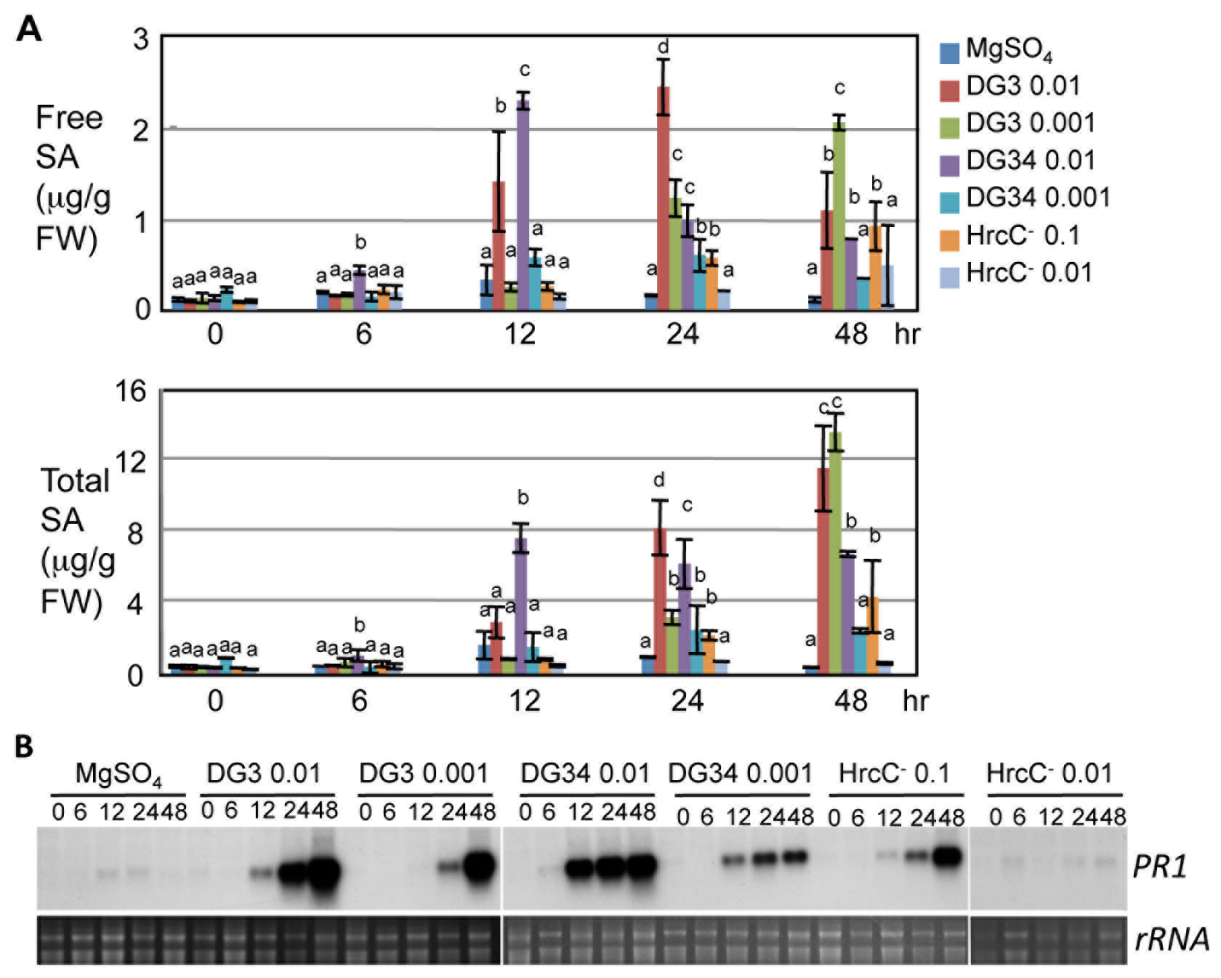
Dynamic changes in SA accumulation and PR1 expression during PTI, ETS, and ETI. The fourth to sixth leaves of 30-day-old Col-0 plants were infected with *Pseudomonas syringae* *pv.* *maculicola* ES4326 strains, DG3 at OD_600_ 0.01 or 0.001, DG34 at OD_600_ 0.01 or 0.001, or HrcC^-^ at OD_600_ 0.1 or 0.01. The infected leaves were collected at the indicated times for SA and RNA analysis. (A) SA quantitation by HPLC analysis. Statistical analysis was performed with one-way ANOVA Fisher’s PLSD tests (StatView 5.0.1). Different letters indicate significant difference among the samples at the same time point (P<0.05). (B) Northern blotting for PR1 expression. Image of rRNA was used for a loading control.

We found that there are kinetic changes in SA accumulation and *PR1* expression, depending on the strains and doses used (Figure 1). Consistent with robust defense triggered by the recognition of avrRpm1 by RPM1 [40], DG34 (0.01)-infected plants showed earliest induction of both free and total SA, beginning at 6 hr post infection (hpi) and reaching the peak at 12 hpi (Figure 1A). Compared with DG34 (0.01), DG3 (0.01) induced delayed but higher SA accumulation with free SA peak at 24 hpi and total SA peak at 48 hpi (Figure 1A). The pattern of *PR1* expression induced by these two strains at OD_600_ 0.01 was consistent with SA accumulation, with an earlier and stronger induction by DG34 (0.01) than by DG3 (0.01) at 6 and 12 hpi but at 48 hpi *PR1* level was higher in tissue infected by DG3 (0.01) than by DG34 (0.01) (Figure 1B). 

Compared with the higher dose of DG34 (0.01), the lower dose of DG34 (0.001) induced lower SA accumulation and *PR1* expression with delayed peaks in the infected plants (Figure 1A and 1B). The lower dose of DG3 (0.001) induced similar levels of SA as DG34 (0.001) before 24 hpi. At 48 hpi, plants infected with DG3 (0.001) showed even higher levels of free and total SA and stronger expression of *PR1* than those infected with DG34 at both doses (0.01 and 0.001) (Figure 1A and 1B). Therefore these results highlight that the differences between ETS and ETI are kinetic and are also dependent on the doses of strains used. 

Compared with DG3 and DG34 strains, HrcC^-^ at both doses induced much weaker defense responses. Only small levels of both free and total SA and *PR1* transcripts were induced at 24 and 48 hpi in plants challenged with HrcC^-^ at OD_600_ 0.1, a concentration that is 10-fold more than the higher dose of DG3 and DG34. HrcC^-^ at OD_600_ 0.01, however, only induced weak expression of *PR1* at 48 hpi (Figure 1A and 1B). Together, these results support the quantitative differences between PTI, ETS, and ETI with PTI being the weakest defense as previously suggested [10]. However, the differences between ETS and ETI are kinetic and dose-dependent. While the induction of SA and *PR1* expression is faster in ETI induced by DG34 (0.01) than in ETS induced by DG3 (0.01), the strength of the induction is actually higher in ETS than ETI at later time points. Such differences between ETS than ETI are largely abolished when the lower dose (0.001) of the strains was used. 

### Dynamic changes in cell death during PTI, ETS, and ETI

Cell death is commonly known to be induced during Arabidopsis-*P. syringae* interactions. It is generally believed that ETI activates stronger and faster cell death than ETS and PTI is only associated with weak cell death. Our analysis of Arabidopsis infected with a higher dose of DG3 or DG34 strain (OD 0.01) supports this notion. We found that DG34 (0.01) induced minor cell death in the infected leaves as early as 6 hpi, which became massive from 24 to 96 hpi (Figure S1). Compared with DG34 (0.01), DG3 (0.01)-induced cell death was much delayed, appearing around 24 hpi and becoming massive at 96 hpi (Figure S1). However, such a difference in cell death induction by the higher dose of DG3 and DG34 strains was much minimized when a lower dose of the strains was used. Both DG34 (0.001) and DG3 (0.001) induced much weaker but comparable cell death in the infected leaves from 24 to 96 hpi (Figure S1, arrows indicate single or small clusters of dead cells). Thus these results further support that the differences between ETS and ETI are dependent on the doses of the strains used, as shown for SA accumulation and *PR1* expression (Figure 1). Since infection with HrcC^-^ at both concentrations (0.1 and 0.01) only induced minor cell death from 24 hpi to 96 hpi (Figure S1), it is possible that effectors are required to activate strong programmed cell death in host cells and the severity of cell death is correlated with the type of effectors (virulence or avirulence) as well as the quantity of the effectors delivered to host cells. 

### Dynamic ROS accumulation and localization during PTI, ETS, and ETI

Oxidative burst is a key signature during host-pathogen interactions [21,22]. However, where and when ROS are produced during PTI, ETS, and ETI have not been well understood. To provide a better understanding of ROS accumulation and localization during PTI, ETS, and ETI, we infected Arabidopsis leaves with the three strains at OD_600_ 0.01 and collected the leaves in a time course (0, 6, 12, 18, 24, and 48 hpi) for fixation in the presence of cerium chloride. Cerium ion reacts with H_2_O_2_ to produce electron-dense insoluble precipitates of cerium perhydroxides [38,39]. The fixed tissue was further embedded and sectioned for transmission electron microscope (TEM) analysis for the presence of electron-dense cerium deposits, an indicative of H_2_O_2_ accumulation. 

**Figure 2:**
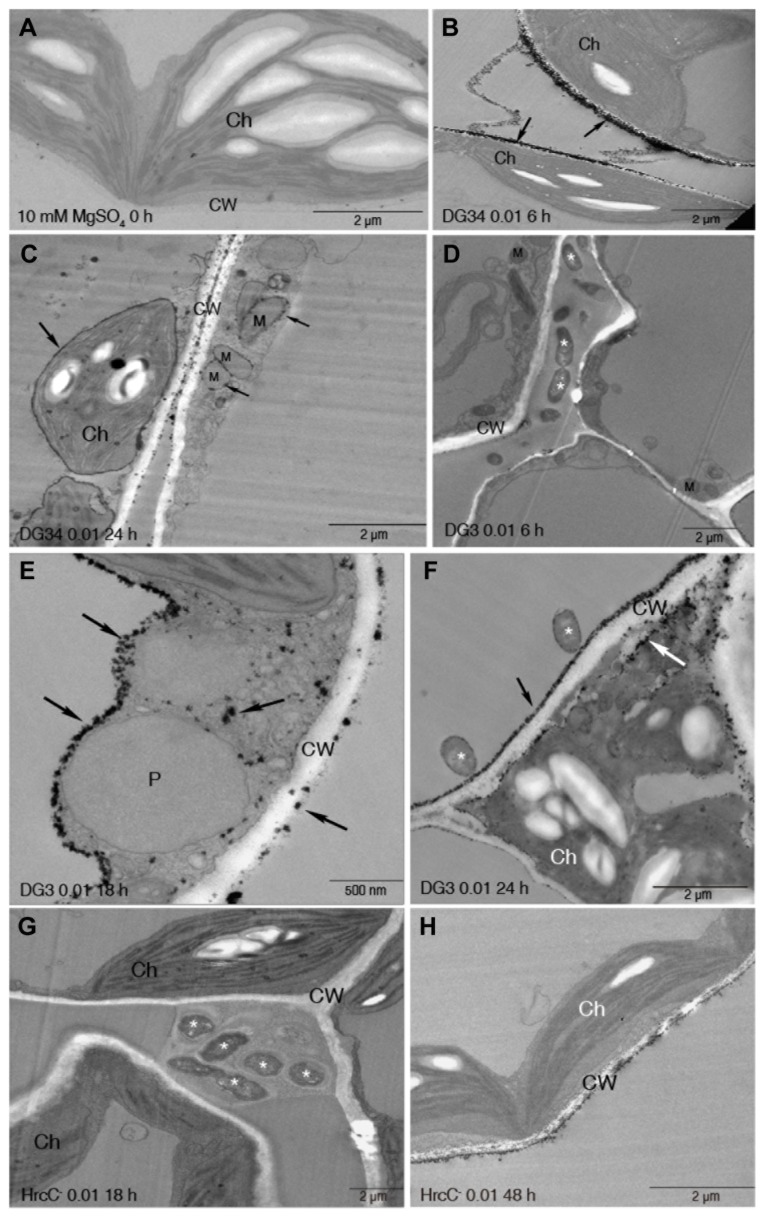
Dynamic ROS accumulation and localization during PTI, ETS, and ETI. The fourth to sixth leaves of 30-day-old plants were infiltrated with *P. syringae* strains at OD_600_ 0.01, using 10 mM MgSO_4_ treatment as a control. The infiltrated leaves were collected at the indicated times and were further cut into 1x2 mm sections. The sections were incubated with freshly prepared 5 mM CeCl_3_ in 50 mM MOPS at pH 7.2 or MOPS without CeCl_3_ for 1 h. The samples were then fixed and processed for TEM imaging. At least six different leaf samples for each treatment were fixed, and six sections were observed in each sample. (A) Cell morphology at 0 h. (B-C) H_2_O_2_ localization at 6 h (B) and 24 h (C) after DG34 inoculation. (D-F) H_2_O_2_ localization at 6 h (D), 18 h (E) and 24 h (F) after DG3 inoculation. (G-H) H_2_O_2_ localization at 18 h (G) and 48 h (H) after HrcC^-^ inoculation. Arrows indicate electron-dense cerium deposits. Asterisks indicate bacteria. Ch, chloroplast; CW, cell wall; M, mitochondrion; P, peroxisome.

Mock-treated leaves did not show cerium deposits (Figure 2A and S2). With DG34 infection, we observed strong cerium deposits initially on the cell wall at 6 hpi (Figure 2B and S3). As infection progressed, cerium deposits were additionally found on the plasma membrane and the outer membranes of the chloroplast and mitochondrion from 24 to 48 hpi (Figure 2C and S3). Compared to DG34, DG3 did not induce detectable cerium deposits in the host cells until 18 hpi (Figure 2D, 2E, and S4). Besides the cell wall, the initial cerium deposits were also found abundant on the tonoplast membrane and in the cytoplasm with DG3 infection at 18 hpi (Figure 2E). At 48 hpi, we also observed electron dense deposits on the mitochondria membrane (Figure S4F). On the other hand, HrcC^-^ infection only induced weak cerium deposits on the cell wall at 48 hpi (Figure 2G, 2H, and S5). These results indicate that the rate of H_2_O_2_ production varies with ETI being the fastest and PTI being the slowest, corroborating previous studies [38,45,46]. While the cell wall is the primary location for initial H_2_O_2_ accumulation during PTI, ETS, and ETI, ETS-induced initial H_2_O_2_ accumulation was also found in intracellular organelles while ETI-induced H_2_O_2_ accumulation was only observed later inside the cell. Therefore, such differential localization and accumulation of H_2_O_2_ suggest that there are different mechanisms of generating H_2_O_2_ during PTI, ETS, and ETI. It is also possible that H_2_O_2_ molecules induced by pathogens are differentially redistributed during PTI, ETS, and ETI, contributing different mechanisms of PTI, ETS, and ETI in the host. 

### Induction of abnormal growths in leaves during late infection

**Figure 3:**
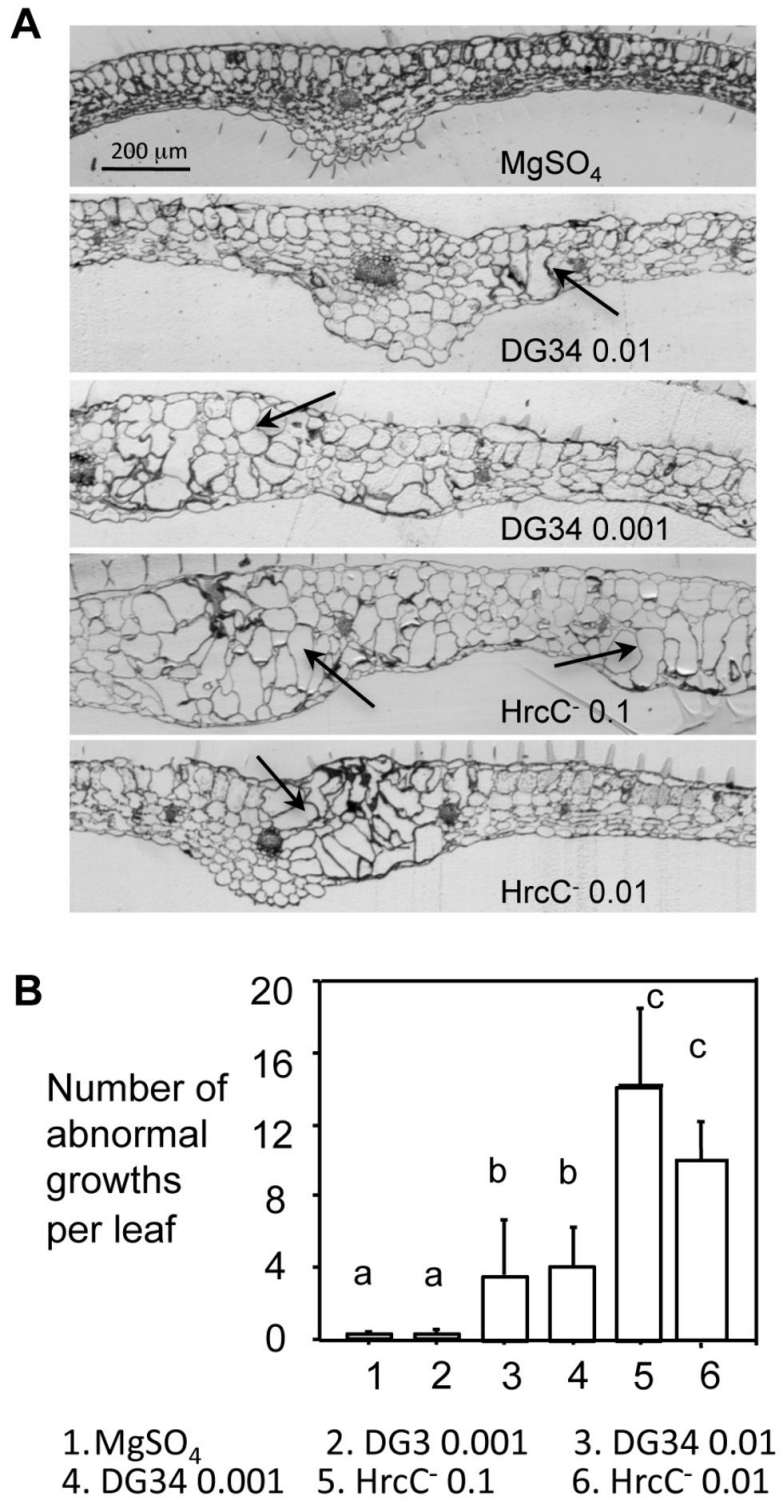
The abnormal growth is mainly induced during PTI. The fourth to sixth leaves of 30-day-old Col-0 plants were infected with *P. syringae* strains and observed for leaf morphology. (A) Pictures of leaf cross sections. Infected leaves were collected at 4 dpi and fixed for embedding with LR White resin. One-micron sections were cut and stained with 1% toluidine blue O for photographing. Leaves infected with DG3 (0.01) were mostly dead at 4 dpi and thus no data is available. Arrows indicate enlarged cells. The size bar represents 200 μm and applies to all images. Each growth (or a protrusion) has multiple enlarged cells. (B) Quantitation of abnormal growths. The number of abnormal growths, appearing to be transparent protrusions on the treated leaves, was counted at 4 dpi with a dissecting microscope. At least 25 leaves from each treatment were used in the counting. Statistical analysis was performed with one-way ANOVA Fisher’s PLSD tests (StatView 5.0.1). Different letters indicate significant difference among the samples (P<0.05).

During the course of the experiments, we carefully examined morphology of the infected leaves. We observed some chlorotic protrusions on the leaves, which began to appear at 3-day post infection (3dpi) and became obvious at 4 dpi (Figure S6A and S6B). We fixed the abnormal growth regions with resin and cut the fixed tissue to semi-thin sections for light microscopy analysis. We found that the abnormal growths consist mainly of enlarged mesophyll cells (Figure 3A, arrows indicate enlarged cells). We also quantified abnormal growth regions by counting the transparent protrusions in the infected leaves, using a dissecting microscope. We found that HrcC^-^-infected leaves showed more protrusions than leaves infected with DG34 (Figure 3B). DG3, on the other hand, did not induce significantly more abnormal growths than the mock treatment. We further fixed and embedded abnormal growth regions for a TEM study. Observations of ultra-thin sections of embedded tissue revealed that the enlarge cells have thicker cell wall than the normal mesophyll cells (Figure S6C).

**Figure 4:**
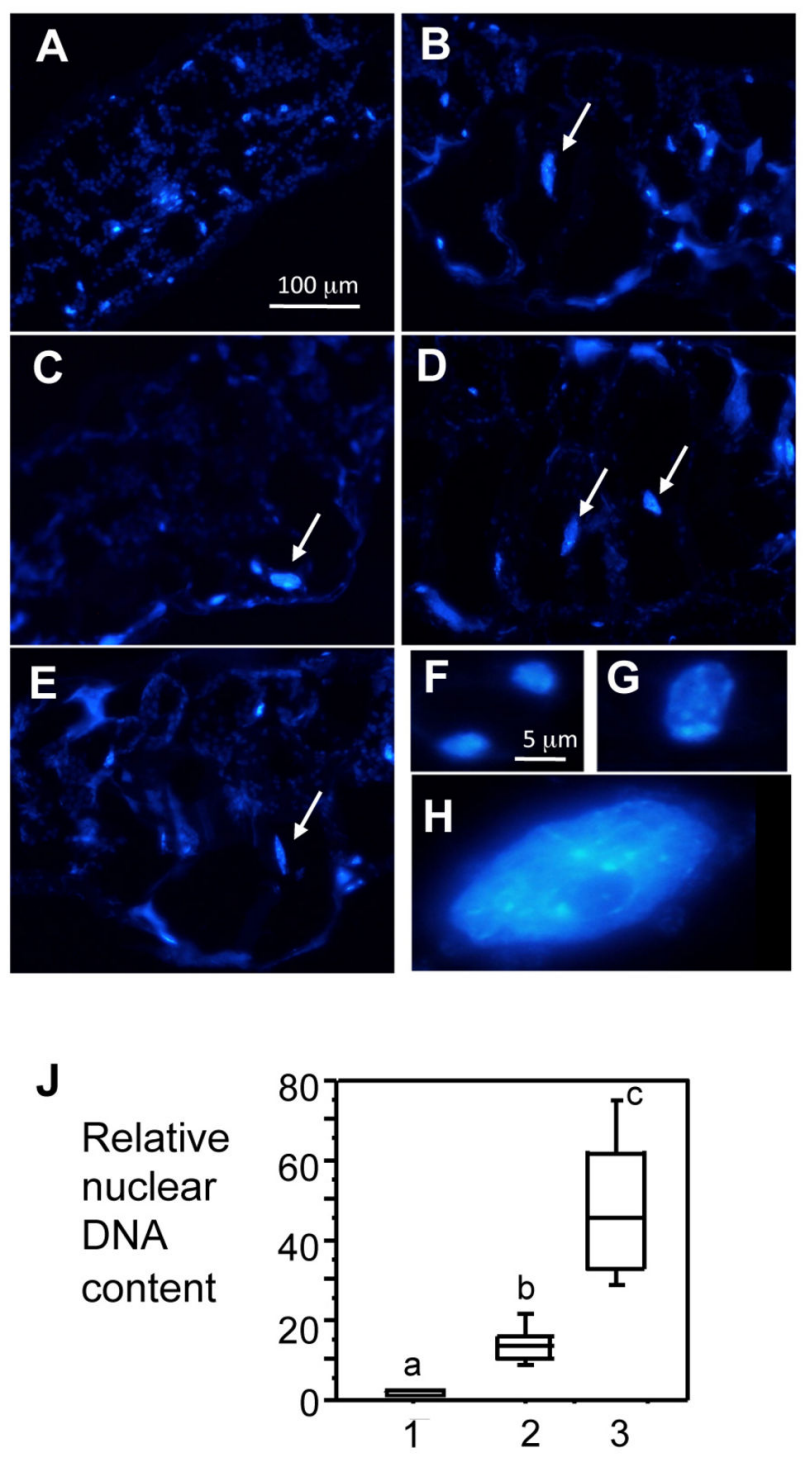
The enlarged cells have increased nuclear DNA content. *P. syringae*- infected leaves were embedded in paraplast and cut into 15 μm sections for DAPI staining. Images of leaf cross-sections stained with DAPI to show nuclei were captured with a constant exposure time, using a Nikon DS cooled camera attached to a compound microscope. The images are from the following treatments: (A) 10 mM MgSO4, (B) DG34 0.01, (C) DG34 0.001, (D) HrcC^-^ 0.1, and (E) HrcC^-^ 0.01. (F) Typical nuclei of guard cells from a mock-treated leaf. Guard cells from leaves infected with DG34 0.01, DG34 0.001, HrcC^-^ 0.1, or HrcC^-^ 0.01 show visually similar nuclei (data not shown). (G) A typical nucleus of a normal mesophyll cell from a mock-treated leaf. Normal sized mesophyll cells from leaves infected with DG34 0.01, DG34 0.001, HrcC^-^ 0.1, or HrcC^-^ 0.01 show visually similar nuclei (data not shown). (H) A typical nucleus of an enlarged mesophyll cell induced by HrcC^-^ 0.01. Enlarged cells from leaves infected with DG34 0.01, DG34 0.001, or HrcC^-^ 0.1 also show large nuclei (data not shown). Arrows indicate the large nuclei in (A) to (E). The size bar in (A) represents 100 μm and applies to panels (A) to (E) while the size bar in (F) represents 5 μm and applies to panels (F) to (H). (J) Relative nuclear DNA content. The average fluorescence of nuclei of guard cells from mock-treated leaves (1) was set as 2C and was used to quantify relative nuclear DNA content of normal mesophyll cells from mock-treated leaves (2) and the enlarged mesophyll cells induced by HrcC^-^ (0.01) (3). At least 60 nuclei were used for each data point. Nuclear DNA contents of guard cells and normal mesophyll cells from HrcC^-^ (0.01)-infected leaves are similar to those of their corresponding cells from mock-treated leaves (data not shown). Statistical analysis was performed with one-way ANOVA Fisher’s PLSD tests (StatView 5.0.1). Different letters indicate significant difference among the samples (P<0.05).

Cell enlargement is often associated with increased nuclear DNA content. To test if this is the case for the enlarged cells, we embedded the tissue showing chlorotic protrusions with paraplast and stained the sections with DAPI to show nuclei [36,47]. Compared with mock-treated leaf cells (Figure 4A), we found that the enlarged cells induced by DG34 (0.01 and 0.001) and HrcC^-^ (0.1 and 0.01) showed much larger nuclei (Figure 4B-4E, arrows indicate large nuclei). Images of typical nuclei from guard cells, mesophyll cells, and large cells were shown side-by-side with a higher magnification to illustrate the size difference (Figure 4F-4H). We further quantified the relative fluorescence unit (RFU) of the stained nuclei with ImageJ (Version 1.45s). Based on the RFU of nuclei and the assumption that the nuclear DNA content of a guard cell is 2C, we derived the relative nuclear DNA content of the enlarged cells. We found that the enlarged cells have an average of 50C nuclear DNA content, much larger than those of the normal mesophyll cells (18C) in mock-treated leaves (Figure 4J). The nuclei contents of guard cells and normal mesophyll cells from infected leaves were comparable to those from the corresponding cells in mock-treated leaves (data not shown). Such an increase in DNA content of the host cells upon infection is possibly due to the activation of endoreplication, a process involving DNA replication without subsequent mitosis [48]. 

### Flg22 but not BTH treatment induces abnormal growth in leaves

**Figure 5:**
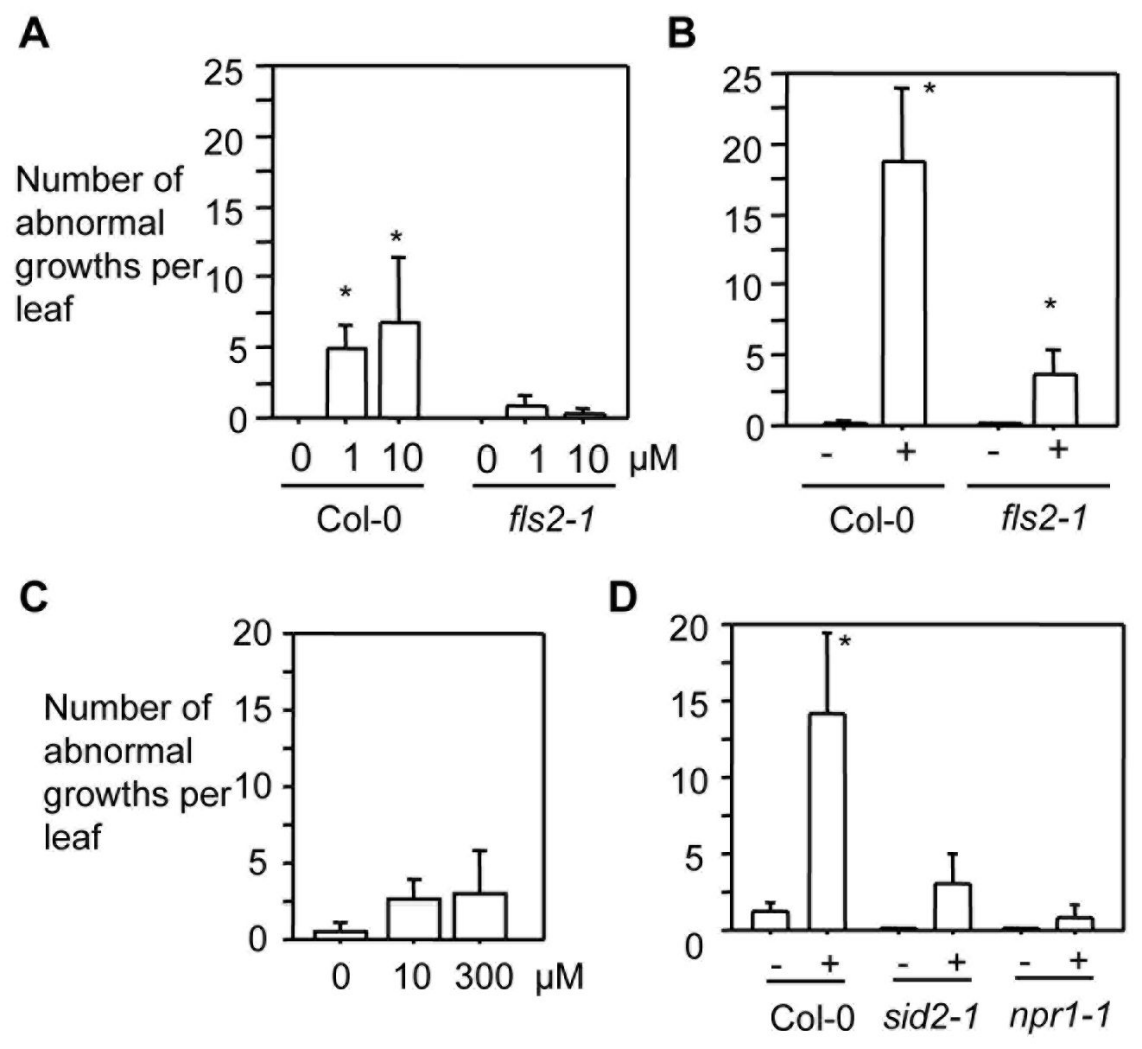
FLS2-mediated signaling but not SA induces cell enlargement in Arabidopsis leaves. Leaves infiltrated with HrcC^-^ (OD_600_ 0.1), flg22 (1 μM or 10 μM), or BTH (10 μM or 300 μM) were quantified for the formation of abnormal growths 4 days post treatment, using a dissecting microscope. (A) Flg22-induced cell enlargement is FLS2-dependent. (B) HrcC^-^ partially requires FLS2 to induce large cells. (C) BTH treatment does not induce abnormal growth. (D) HrcC^-^-induced abnormal growth requires SID2 and NPR1. For quantification of abnormal growths, at least 25 leaves from 12 plants were used for each BTH treatment or bacterial infection and 18 leaves from 7 plants were used for each flg22 treatment. Statistical analysis was performed with one-way ANOVA Fisher’s PLSD tests (StatView 5.0.1). Asterisks in (A), (B), and (D) indicate significant difference between treatments of the same genotype (P<0.05).

The fact that the HrcC^-^ strain induces more abnormal growths than both DG3 and DG34 strains suggests that PTI plays a major role in activating cell growth. To further test this, we infiltrated plants with flg22, a 22-aa synthetic peptide derived from the conserved sequence of flagellin proteins of *P. syringae* that is commonly used to elicit PTI in plants [4]. We found that both doses of flg22 (1 μM and 10 μM) induced significantly more abnormal growths in leaves (Figure 5A and S7A). When disrupting flg22 perception with the *fls2-1* mutation [4], the formation of enlarged cells was abolished. However, HrcC^-^ (0.1)-induced abnormal growths were drastically reduced but not abolished in *fls2-1* (Figure 5B and S7B). Therefore, we conclude that flg22-FLS2 recognition triggered signaling is one of the ways to activate cell growth. Other PAMPs could also play a role in affecting host cell fate determination. 

SA is a signaling molecule critical for PTI and ETI [49,50]. We found that infiltration of Col-0 with BTH (an SA agonist) did not induce significantly more abnormal growths in the leaves, compared with mock-treated Col-0 (Figure 5C). However, disrupting SA biosynthesis by *sid2-1* or SA signaling by *npr1-1* compromised the formation of enlarged cells in the presence of HrcC^-^ (0.1) (Figure 5D). These data suggest that SA and/or SA signal are necessary but not sufficient to induce large cell formation in Arabidopsis. 

## Discussion

In this report, we systematically examined phenotypes associated with PTI, ETS, and ETI in a time course, including SA accumulation, *PR1* expression, cell death formation, and H_2_O_2_ production/localization. Our data show dynamic changes of these defense related phenotypes during PTI, ETS, and ETI. They also suggest that the differences between ETS and ETI are dependent on the doses of the strains used. While our data corroborate the quantitative nature of the biological system [3,10], they have also revealed the qualitative differences among PTI, ETS, and ETI, in terms of H_2_O_2_ localization. Interestingly, we also observed a differential regulation of cell fate during PTI, ETS, and ETI. Thus, the biological system is complicated; it involves not only a large set of common phenotypes induced by various pathogens with different quantities and kinetics, but also distinct responses to specific pathogens. 

It is generally believed that PTI is a slow and low mode of defense in the host, ETI is an amplified version of PTI, and host defense is suppressed during ETS. Consistent with this notion, we found that the rate of SA accumulation, *PR1* expression, and cell death, is faster during ETI than during ETS, when we used a higher dose (OD 0.01) of DG3 and DG34 to infect plants. However, with a lower dose (OD 0.001) of the strains, we found that ETI and ETS behave grossly similarly in terms of SA accumulation, *PR1* expression, and cell death at early time points (Figure 1 and S1). Therefore these results indicate that the differences between ETI and ETS are dependent on the doses of strains used. Our data further show that the differences between ETI and ETS are kinetic. The levels of SA and *PR1* transcripts and the severity of cell death are comparable between ETI (induced by DG34 0.01) and ETS (induced by DG3 0.01) or are even higher during ETS than during ETI at later time points (Figure 1 and S1). Such dynamic and dose-dependent defense responses suggest that cautions should be taken when comparing plants for their defense phenotypes. For instance, one should use different doses of pathogens to infect plants and sample infected tissue at different time points in order to detect differences in plant defense responses. Our careful time-course analysis of defense related phenotypes on SA accumulation, *PR1* expression, cell death formation, and H_2_O_2_ localization/accumulation could provide a framework to guide the design of time-series experiments to compare defense mutants with wild type for their PTI, ETS, and ETI responses. 

Oxidative burst is critical for host-pathogen interactions [21,22]. Many prior studies used methods involving dye staining [51–53], fluorescence [51–54], and/or chemiluminescence [55] to detect ROS production. These methods are based on the activities of oxidase enzymes, such as peroxidase and NADPH oxidase, and therefore indirectly measure the levels of ROS *in planta*. Although providing relative quantitative information on ROS production, these methods do not offer precise subcellular localization of ROS, which could play a critical role in defense signal activation and transduction. The cerium chloride-based method, on the other hand, detects via TEM the electron dense precipitates resulting from the reaction of CeCl_3_ with H_2_O_2_, thus allowing a direct detection of H_2_O_2_ at the organelle levels. This method has been used in several plant-pathogen systems, such as Arabidopsis and lettuce-*P. syringae* [38,56], French bean–*Xanthomonas* [57], tomato/bean-*Botrytis cinerea* [58], legume-*Rhizobia* [59], tomato-nematode [45], and *Solanum*-Potato virus Y [60], and has provided spatial information of H_2_O_2_ accumulation in these plant pathosystems. 

We used this method for the first time to systematically examine when and where ROS is produced during PTI, ETS, and ETI. Our data have revealed temporal and spatial resolution of H_2_O_2_ localization during Arabidopsis-*P. syringae* interactions. We show that ETI is associated with much faster H_2_O_2_ accumulation than ETS, which is consistent with previous studies [38,45,46]. Our data also show spatial difference in H_2_O_2_ accumulation, with ETI-induced H_2_O_2_ initially on the cell wall then inside of the cell and ETS-induced H_2_O_2_ beginning both inside and outside of the cell. H_2_O_2_ may contribute to cell death caused by DG34 and DG3 since the induction of H_2_O_2_ precedes the timing of massive cell death in the infected tissue (Figure S1 and 2). PTI-induced H_2_O_2_, on the other hand, accumulated much slowly and was only limited to the cell wall. Such temporal and spatial differences in H_2_O_2_ accumulation might reflect different mechanisms of plant resistance during PTI, ETS, and ETI. 

Pathogen-induced programmed cell death has been the focus in the studies of host-pathogen interactions [61,62]. However, the change of host cell growth upon pathogen infection has been largely overlooked. Some animal pathogens are known to interfere with the host cell cycle machinery, activating cell growth (cell division and/or cell enlargement) and sometimes leading to tumorigenesis in animals [63,64]. Pathogen-triggered cell growth has also been documented in some plants. For instance, nematodes induce host plants to form large cells, either resulting from fusion of the infected cells with its neighboring cells or from cell enlargement [23]. The fungal pathogen powdery mildew was also shown to induce cell enlargement in Arabidopsis [24]. These enlarged cells have increased nuclear DNA content, suggesting an activation of endoreplication [23,24,65]. In addition, bacteria, such as *Xanthomonas* and *Agrobacterium tumefaciens*, can induce gall-like abnormal growths in their specific hosts, which could result from host cell enlargement and/or cell division [26,27,66]. However, the mechanisms underlying host cell fate control upon pathogen infection have not been fully understood.

Here we show that *P. syringae* also induces abnormal growths in Arabidopsis, which consist of enlarged mesophyll cells (Figure 3). The fact that the HrcC^-^ strain induces more abnormal growths than both DG3 and DG34 strains suggests that PTI plays a major role in regulating cell growth. Consistent with this notion, our data further show that flg22- and perhaps also other PAMPs-induced PTI could act together to induce cell fate change in the host (Figure 5A and B). Bacterial effectors were also reported to control host cell fate [25,27,67–70]. Our data show that only the avirulent (DG34) but not the virulent *P. syringae* strain (DG3) induced minor abnormal growths in Arabidopsis although both strains induced massive cell death. These results highlight a possibility that some avirulence effectors promote cell growth in the host while some virulence effectors play a suppressing role. It would be useful to identify additional PAMPs and the effectors of *P. syringae* that regulate cell fate determination in Arabidopsis in order to better understand the molecular mechanisms underlying cell fate control during Arabidopsis-*P. syringae* interactions. 

How are the signals from pathogens, such as bacterial flagellin, other PAMPs, and/or effectors, are transmitted to regulate host cell growth? One possibility is to perturb cell cycle progression, resulting in endoreplication and subsequently enlarged cells. Indeed, several cell cycle related genes are activated during host-pathogen interactions [13,23,24,36,65]. In addition, several components of Anaphase Promoting Complex (APC) that represents a check point of cell cycle progression, were recently shown to play a role in defense control [71]. Consistent with this speculation, we show here that enlarged cells have increased DNA content, a likely consequence of endoreplication. Fusion of plant cells in the infected region could also result in an increase in DNA content of the enlarged cells. However, we have not observed any morphological evidence to illustrate the cell fusion process. 

Pathogens could also regulate cell fate via manipulating host hormones. For instance, *A. tumefaciens* harbors genes encoding enzymes for the biosynthesis of cytokinins and auxin [25], which can be expressed in the host to perturb hormonal profile and subsequently induce crown galls in the infected plants. Manipulating SA signaling is also a potential way to affect host cell fate. SA-dependent cell fate change has been reported in several defense mutants with lesion mimic phenotypes [36,72,73]. Here we show that activation of SA signaling is necessary but not sufficient to induce cell enlargement in Arabidopsis. Thus, SA and an additional signal(s), potentially induced by pathogen infection, are required to regulate host cell fate change. It would be interesting to further elucidate what these additional signaling components are and how they affect host cell fate determination during Arabidopsis-*P. syringae* interaction. 

Why should hosts form enlarged cells upon pathogen attack? The enlarged cells often have higher nuclear DNA content (this study and [23,24]) and perhaps also increased metabolic activities [48,74]. Thus they might confer a higher capacity to plant cells to respond to the accumulation of mutations and/or adverse stresses or to provide better nutrient reservoir for plant cells to increase their sizes. On the other hand, the rich nutrient reservoir can be hijacked by some pathogens that feed on these cells. Thus, these large cells can be the battleground during plant-pathogen interactions. The formation of large cells has been suggested as a susceptible response of hosts in several plant pathosystems [23–26,70]. Here we show that during Arabidopsis-*P. syringae* interactions, abnormal growths are induced more abundantly during PTI and ETI but not during ETS (Figure 3). In particular, we found that the large cells have thicker cell wall, possibly imposing a physical barrier to prevent further infection of pathogens (Figure S6C). Such cell wall thickening of large cells induced by PTI is consistent with previous studies showing that PAMPs induce expression of genes involved in cell secretion and cell wall modification [13,44]. Therefore, we propose that large cell formation might be a resistant response during Arabidopsis-*P. syringae* interactions. There are still many unanswered questions regarding cell fate control during host-pathogen interactions, a topic that warrants further investigations in order to yield a better understanding of mechanisms of cell fate control and disease resistance in plants.

## Supporting Information

Figure S1 **Dynamic changes in cell death during PTI, ETS, and ETI.** The fourth to sixth leaves of 30-day-old Col-0 plants were infected with *P. syringae* strains as described in Figure 1. The infected leaves were collected at the indicated times for trypan blue staining to visualize cell death. Images of the stained leaves were taken with a CCD camera connected with a Leica dissecting microscope. The scale bar represents 0.5 mm and applies to all images. Note massive cell death in leaves infected with DG3 0.01 at 48 and 96 hpi or with DG34 0.01 at 24, 48, and 96 hpi. Arrows indicate minor cell death (single dead cells or small clusters of dead cells) in the infected leaves. No cell death was observed in mock-treated leaves.
(TIF)

Figure S2 **No H_2_O_2_ is detected in mock-treated leaves.** (A-F) Cerium staining to detect H_2_O_2_ localization in Col-0 leaves at the indicated times after 10 mM MgSO_4_ treatment. Note the lack of cerium deposits at all times. Ch, chloroplast; CW, cell wall; M, mitochondrion. Size bars represent 2 μm in all images.
(TIF)

Figure S3 **H_2_O_2_ detection in leaves infected with DG34.** (A-H) Cerium staining to detect H_2_O_2_ localization in Col-0 leaves at the indicated times after DG34 inoculation (OD_600_=0.01). Note that cell wall apposition (CWA) with electron-dense cerium deposits (arrows) was found as early as 6 hpi (A). Major cerium deposits were localized on cell wall at 6-18 hpi (B-D). During 24-48 hpi, H_2_O_2_ was also found on the plasma membrane (E), outer membranes of the chloroplast and mitochondrion (F-H). Ch, chloroplast; CW, cell wall; CWA, cell wall apposition; M, mitochondrion.
(TIF)

Figure S4 **H_2_O_2_ detection in leaves infected with DG3.** (A-F) Cerium staining to detect H_2_O_2_ localization in Col-0 leaves at the indicated times after DG3 inoculation (OD_600_=0.01). Note no cerium deposits were observed at the early times (6-12 hpi) (A-C). Drastic H_2_O_2_ production (arrows) was detected in the tonoplast and cytosol (D) as well as on the cell wall (D-E) between 18-24 hpi. At 48 hpi, cerium deposits were also found on outer mitochondrial membrane (F). Asterisks indicate bacteria. Ch, chloroplast; CW, cell wall; M, mitochondrion; P, peroxisome.
(TIF)

Figure S5 **H_2_O_2_ detection in leaves infected with HrcC^-^.** (A-E) Cerium staining to detect H_2_O_2_ localization in Col-0 leaves at the indicated times after HrcC^-^ inoculation (OD_600_=0.01). Note the lack of H_2_O_2_ at the early times (6 to 24 hpi) (A-D). Weak cerium deposits on the cell wall were found at 48 hpi (E). White asterisks indicate bacteria. Ch, chloroplast; CW, cell wall; M, mitochondrion.
(TIF)

Figure S6 **Abnormal growths are induced by *P. syringae* infection.** (A) Leaf hand-sections. Note a chlorotic protrusion in an HrcC^-^ (0.01)-infected leaf (arrow) but not in a mock-treated leaf. Similar protrusions were seen in leaves infected with HrcC^-^ (0.1), DG34 (0.01), and DG34 (0.01) (Data not shown). (B) Images of the abaxial side of leaves. The abaxial side of a mock-treated leaf (top) or an HrcC^-^ (0.01)-infected leaf (bottom) was photographed with a dissecting microscope connected with a camera. Arrows indicate abnormal growths in the HrcC^-^ (0.01)-infected leaf but not in mock-treated leaf. Note the change of leaf color due to the effect of light. (C) Large cells induced by *P. syringae* infection show thicker cell wall. Infected leaves were fixed and embedded for TEM observation. Note cell wall thickening of a typical large cell from a HrcC^-^-infected leaf (0.01) (right panel), compared with a typical mesophyll cell from a mock-treated leaf (left panel). Large cells induced by DG34 (0.01) have similar cell wall thickening as the large cell shown and mesophyll cells of a normal size from infected leaves have similar cell wall as the mesophyll cell shown (data not shown).
(TIF)

Figure S7 **FLS2-mediated signaling induces cell enlargement in Arabidopsis leaves.** The fourth to sixth leaves of 30-day-old Col-0 plants were infiltrated with HrcC^-^ (OD_600_ 0.1), flg22 (1 μM or 10 μM), or mock solutions (10 mM MgSO_4_ for HrcC^-^ and water for flg22). The infiltrated leaves were collected at 4 dpi and fixed for embedding with LR White resin. One-micron sections were cut and stained with 1% toluidine blue O for photographing, using a camera connected to a Leica dissecting microscope. (A) Flg22-induced cell enlargement is FLS2-dependent. (B) HrcC^-^ partially requires FLS2 to induce large cells. Arrows indicate enlarged cells. The size bar represents 200 μm and applies to all images.
(TIF)
