# Supplementary figures and images for: Dynamics of Defense Responses and Cell Fate Change during Arabidopsis-Pseudomonas syringae Interactions
Source: PLoS One. 2013 Dec 11;8(12):e83219. doi: 10.1371/journal.pone.0083219 (PMC3859648; doi:10.1371/journal.pone.0083219)

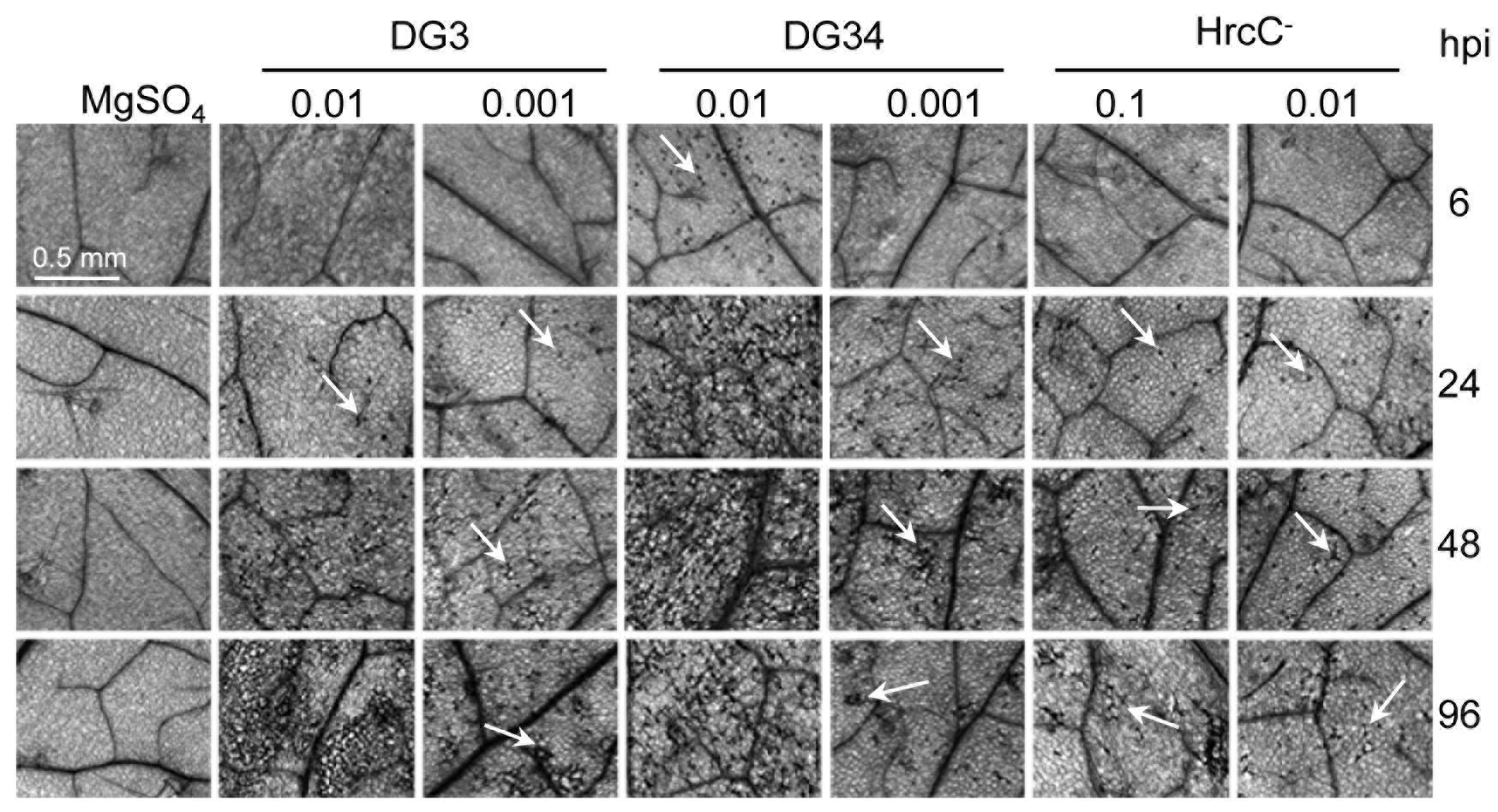

Supplement: Figure S1 — Dynamic changes in cell death during PTI, ETS, and ETI. The fourth to sixth leaves of 30-day-old Col-0 plants were infected with P. syringae strains as described in Figure 1. The infected leaves were collected at the indicated times for trypan blue staining to visualize cell death. Images of the stained leaves were taken with a CCD camera connected with a Leica dissecting microscope. The scale bar represents 0.5 mm and applies to all images. Note massive cell death in leaves infected with DG3 0.01 at 48 and 96 hpi or with DG34 0.01 at 24, 48, and 96 hpi. Arrows indicate minor cell death (single dead cells or small clusters of dead cells) in the infected leaves. No cell death was observed in mock-treated leaves. (TIF) [file pone.0083219.s001.tif]

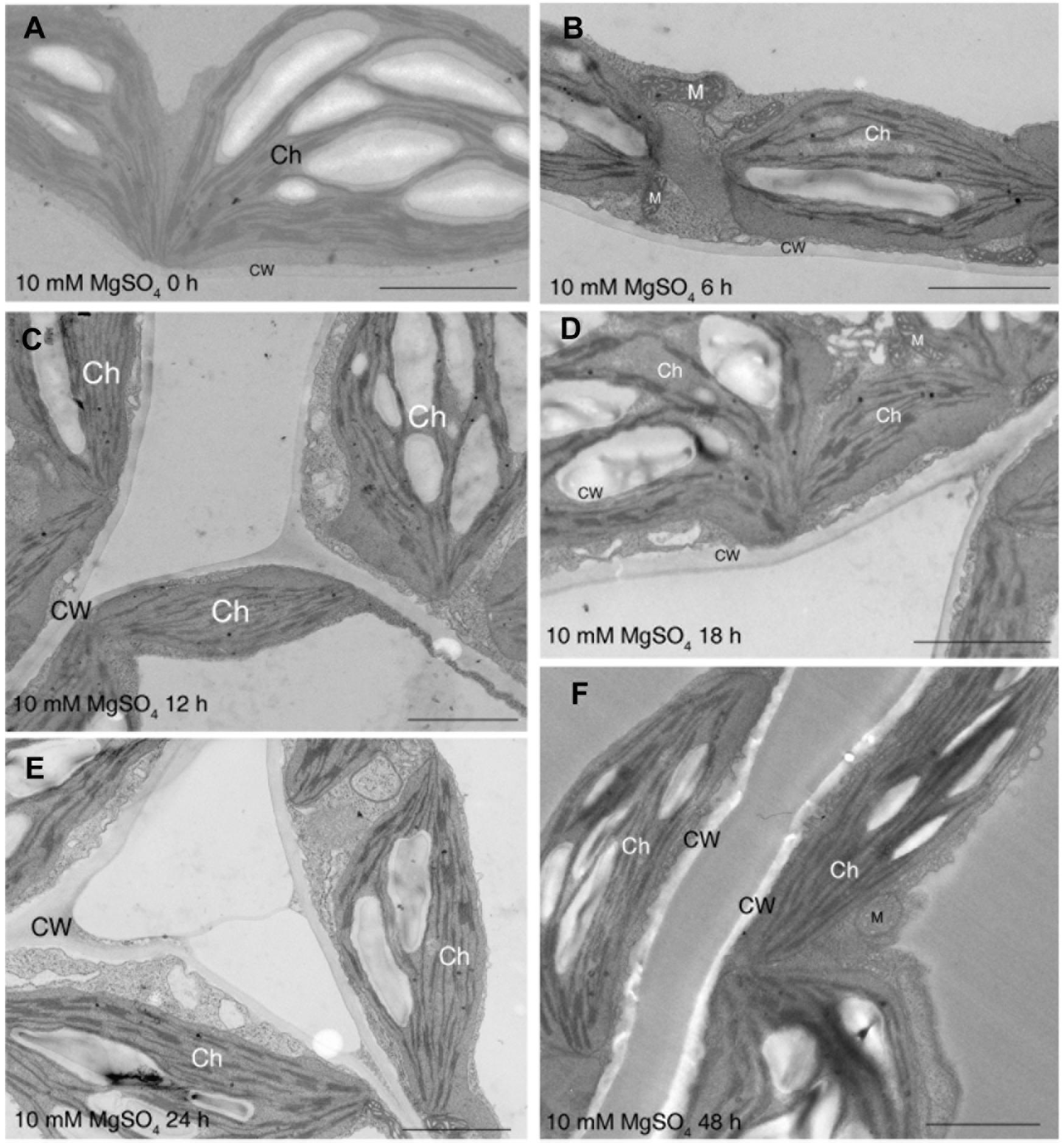

Supplement: Figure S2 — No H2O2 is detected in mock-treated leaves. (A-F) Cerium staining to detect H2O2 localization in Col-0 leaves at the indicated times after 10 mM MgSO4 treatment. Note the lack of cerium deposits at all times. Ch, chloroplast; CW, cell wall; M, mitochondrion. Size bars represent 2 μm in all images. (TIF) [file pone.0083219.s002.tif]

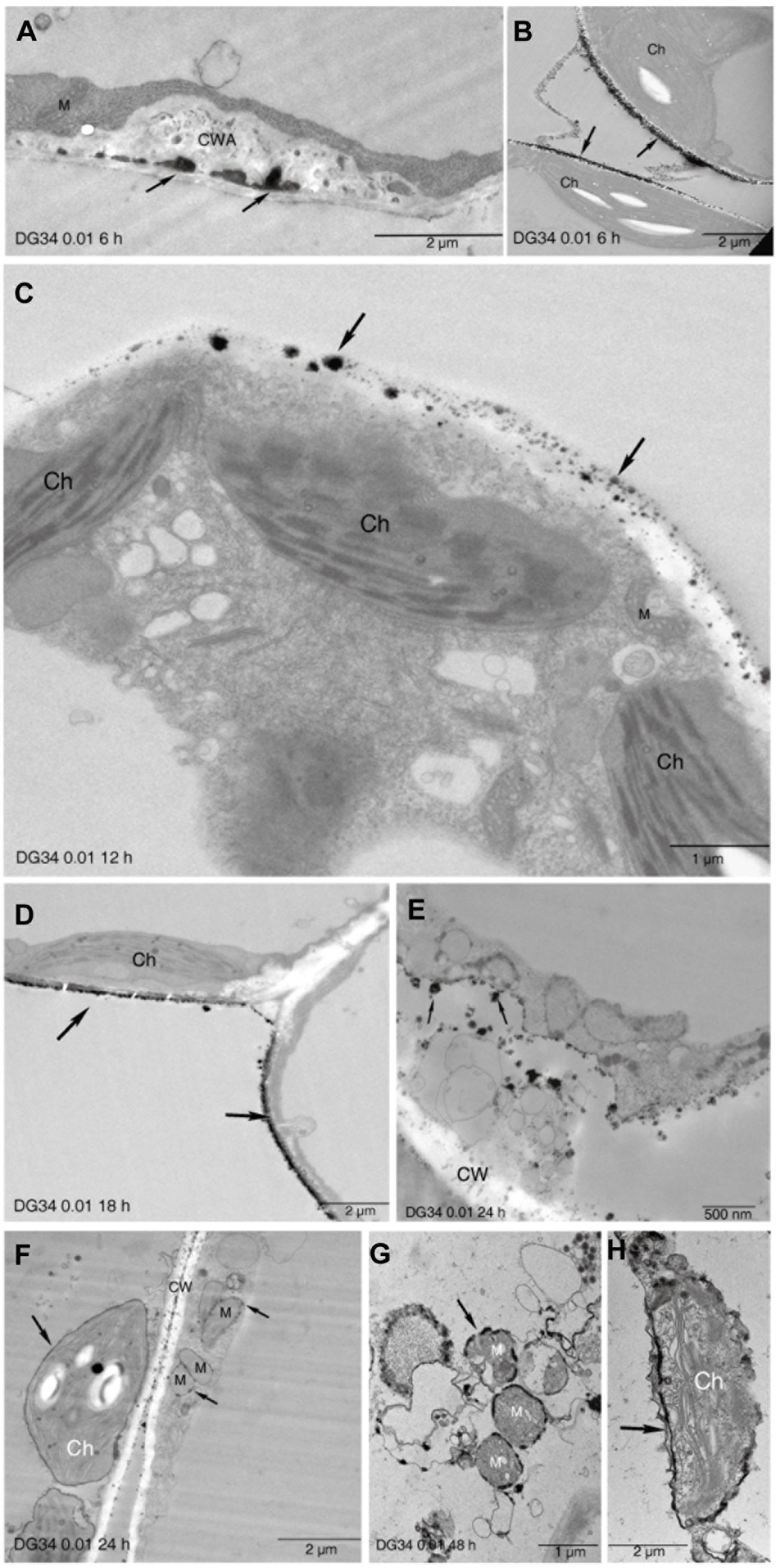

Supplement: Figure S3 — H2O2 detection in leaves infected with DG34. (A-H) Cerium staining to detect H2O2 localization in Col-0 leaves at the indicated times after DG34 inoculation (OD600=0.01). Note that cell wall apposition (CWA) with electron-dense cerium deposits (arrows) was found as early as 6 hpi (A). Major cerium deposits were localized on cell wall at 6-18 hpi (B-D). During 24-48 hpi, H2O2 was also found on the plasma membrane (E), outer membranes of the chloroplast and mitochondrion (F-H). Ch, chloroplast; CW, cell wall; CWA, cell wall apposition; M, mitochondrion. (TIF) [file pone.0083219.s003.tif]

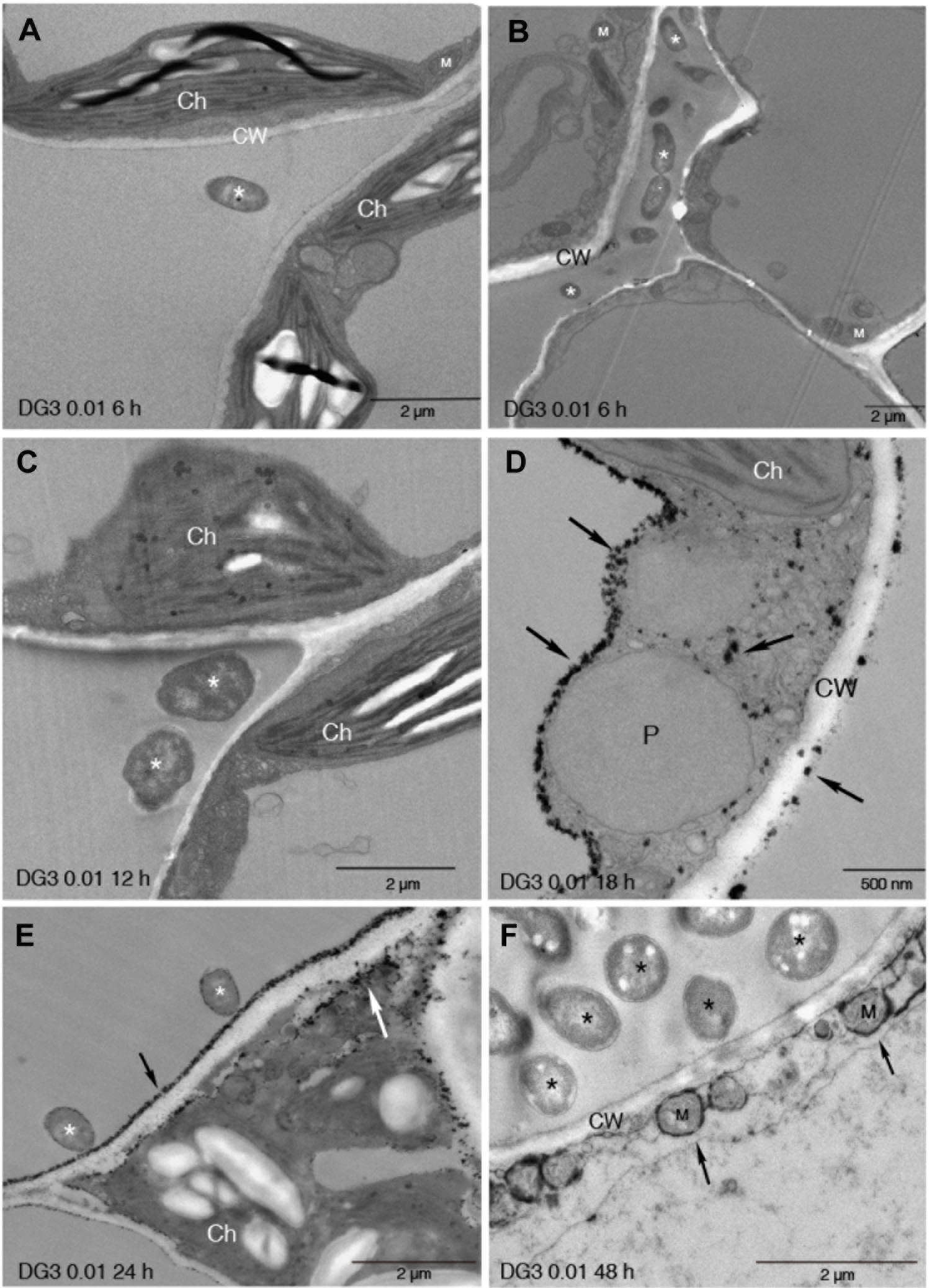

Supplement: Figure S4 — H2O2 detection in leaves infected with DG3. (A-F) Cerium staining to detect H2O2 localization in Col-0 leaves at the indicated times after DG3 inoculation (OD600=0.01). Note no cerium deposits were observed at the early times (6-12 hpi) (A-C). Drastic H2O2 production (arrows) was detected in the tonoplast and cytosol (D) as well as on the cell wall (D-E) between 18-24 hpi. At 48 hpi, cerium deposits were also found on outer mitochondrial membrane (F). Asterisks indicate bacteria. Ch, chloroplast; CW, cell wall; M, mitochondrion; P, peroxisome. (TIF) [file pone.0083219.s004.tif]

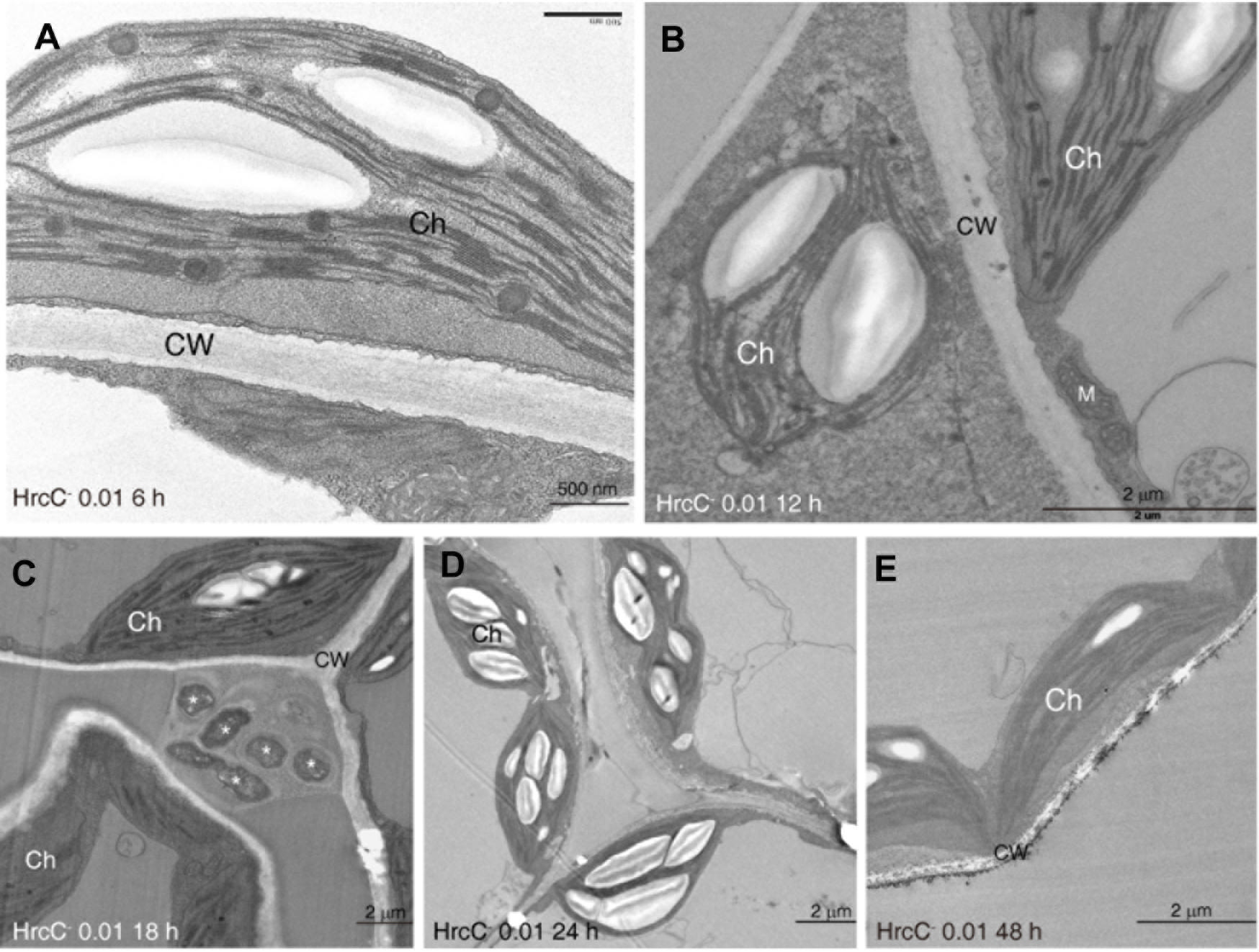

Supplement: Figure S5 — H2O2 detection in leaves infected with HrcC-. (A-E) Cerium staining to detect H2O2 localization in Col-0 leaves at the indicated times after HrcC- inoculation (OD600=0.01). Note the lack of H2O2 at the early times (6 to 24 hpi) (A-D). Weak cerium deposits on the cell wall were found at 48 hpi (E). White asterisks indicate bacteria. Ch, chloroplast; CW, cell wall; M, mitochondrion. (TIF) [file pone.0083219.s005.tif]

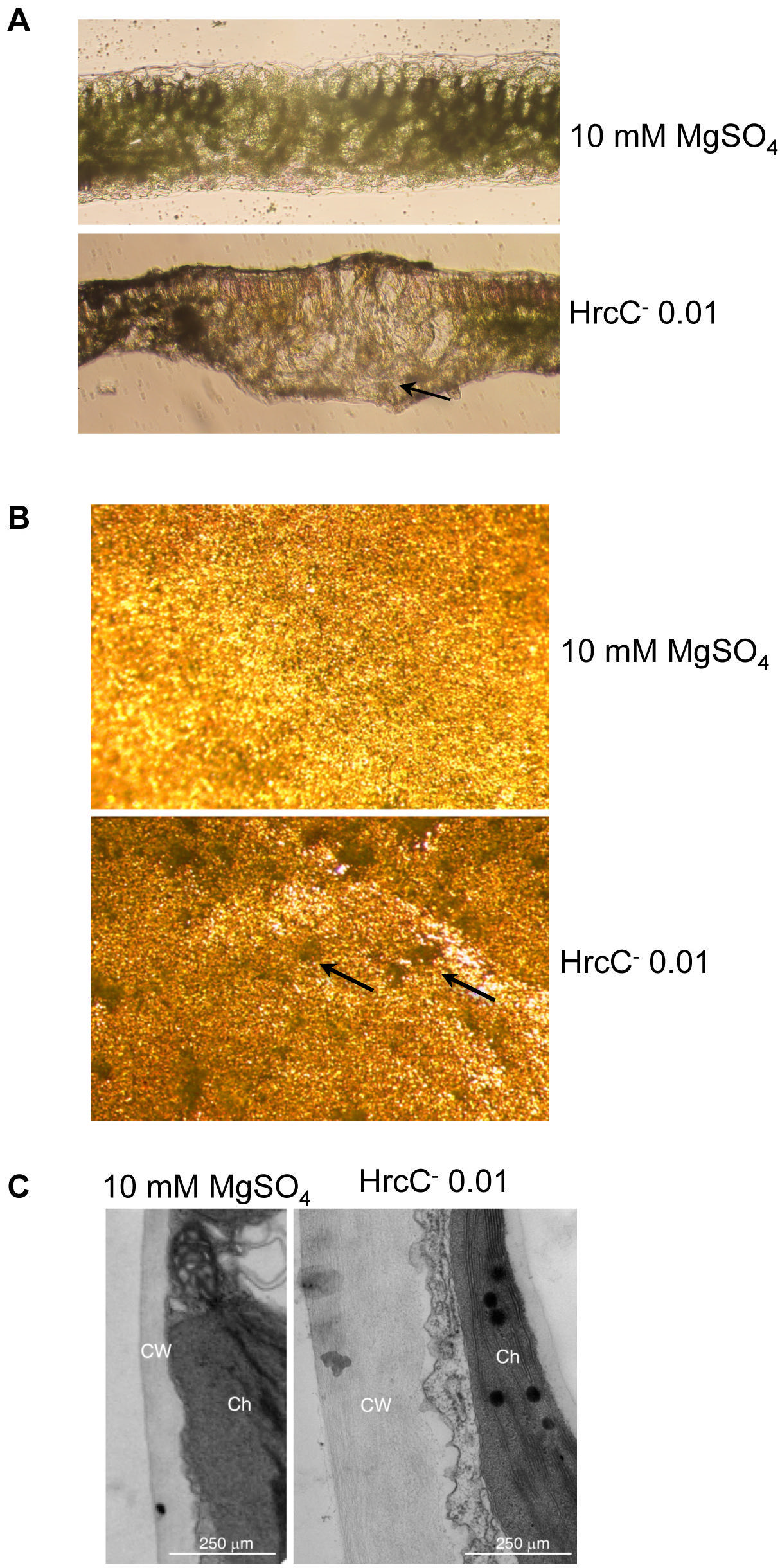

Supplement: Figure S6 — Abnormal growths are induced by P. syringae infection. (A) Leaf hand-sections. Note a chlorotic protrusion in an HrcC- (0.01)-infected leaf (arrow) but not in a mock-treated leaf. Similar protrusions were seen in leaves infected with HrcC- (0.1), DG34 (0.01), and DG34 (0.01) (Data not shown). (B) Images of the abaxial side of leaves. The abaxial side of a mock-treated leaf (top) or an HrcC- (0.01)-infected leaf (bottom) was photographed with a dissecting microscope connected with a camera. Arrows indicate abnormal growths in the HrcC- (0.01)-infected leaf but not in mock-treated leaf. Note the change of leaf color due to the effect of light. (C) Large cells induced by P. syringae infection show thicker cell wall. Infected leaves were fixed and embedded for TEM observation. Note cell wall thickening of a typical large cell from a HrcC--infected leaf (0.01) (right panel), compared with a typical mesophyll cell from a mock-treated leaf (left panel). Large cells induced by DG34 (0.01) have similar cell wall thickening as the large cell shown and mesophyll cells of a normal size from infected leaves have similar cell wall as the mesophyll cell shown (data not shown). (TIF) [file pone.0083219.s006.tif]

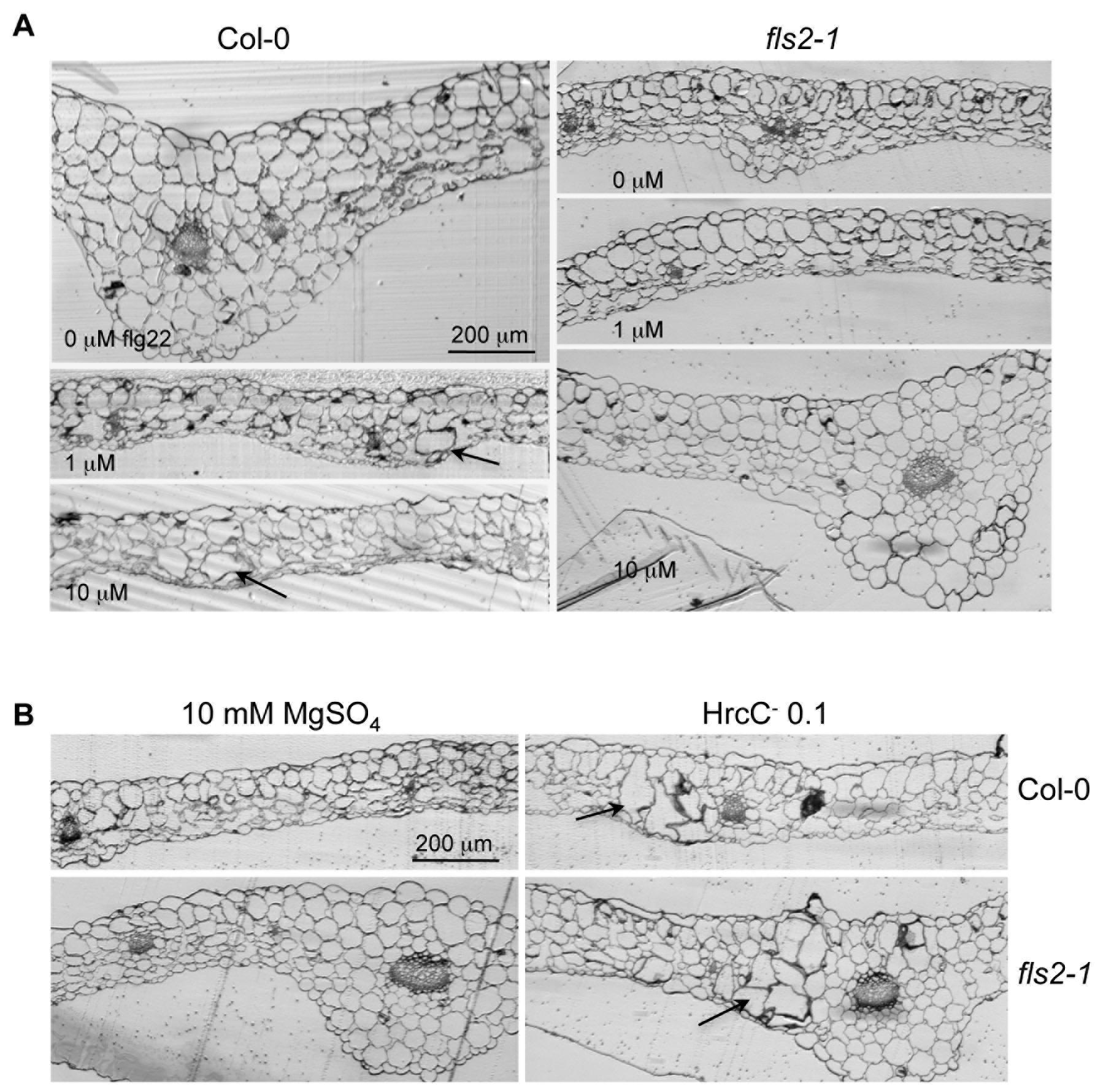

Supplement: Figure S7 — FLS2-mediated signaling induces cell enlargement in Arabidopsis leaves. The fourth to sixth leaves of 30-day-old Col-0 plants were infiltrated with HrcC- (OD600 0.1), flg22 (1 μM or 10 μM), or mock solutions (10 mM MgSO4 for HrcC- and water for flg22). The infiltrated leaves were collected at 4 dpi and fixed for embedding with LR White resin. One-micron sections were cut and stained with 1% toluidine blue O for photographing, using a camera connected to a Leica dissecting microscope. (A) Flg22-induced cell enlargement is FLS2-dependent. (B) HrcC- partially requires FLS2 to induce large cells. Arrows indicate enlarged cells. The size bar represents 200 μm and applies to all images. (TIF) [file pone.0083219.s007.tif]
